# Deep learning based topic and sentiment analysis: COVID19 information seeking on social media

**DOI:** 10.1007/s13278-022-00917-5

**Published:** 2022-07-25

**Authors:** Md Abul Bashar, Richi Nayak, Thirunavukarasu Balasubramaniam

**Affiliations:** Queensland University of Technology, Brisbane, Queensland Australia

**Keywords:** COVID19, Sentiment analysis, Topic analysis, Impact analysis, Informed machine learning, Deep learning, Neural topic modelling, Dynamic topic modelling, SBS

## Abstract

Social media platforms have become a common place for information exchange among their users. People leave traces of their emotions via text expressions. A systematic collection, analysis, and interpretation of social media data across time and space can give insights into local outbreaks, mental health, and social issues. Such timely insights can help in developing strategies and resources with an appropriate and efficient response. This study analysed a large Spatio-temporal tweet dataset of the Australian sphere related to COVID19. The methodology included a volume analysis, topic modelling, sentiment detection, and semantic brand score to obtain an insight into the COVID19 pandemic outbreak and public discussion in different states and cities of Australia over time. The obtained insights are compared with independently observed phenomena such as government-reported instances.

## Introduction

An outbreak of infectious diseases such as COVID19 has a devastating impact on society with severe socio-economic consequences. The COVID19 pandemic has already caused the largest global recession in history; global stock markets have crashed, travel and trade industries are losing billions, schools are closed, and health care systems are overwhelmed. Mental health and social issues creep up as people fear catching the disease or losing loved ones, as they lose jobs, or as they are required to stay in isolation.

An insight into an outbreak is essential for controlling infectious diseases and identifying subsequent mental and social issues Al-garadi et al. (2016). This will help in reducing costs to the economy over the long term and bringing harmony to the society. Especially, early detection helps in placing strategies and resources for an appropriate and efficient response. On social media, people discuss things that they observe in community Al-garadi et al. (2016). They leave traces of their emotions via text expressions Gkotsis et al. (2017). A systematic collection, analysis, and interpretation of social media data can give insight into an outbreak. Twitter is one of the most popular micro-blogging social media websites where users express their thoughts and opinions on real-world events Dahal et al. (2019). Social scientists have used tweet datasets for various purposes such as investigating public opinion of Hurricane Irene Mandel et al. (2012) and election result prediction Tumasjan et al. (2004).

The spatio-temporal texts collected from Sina-Weibo (Twitter alike microblogging system in China) was analysed to understand public opinions on COVID19 related topics Han et al. (2020). They used topic modelling technique Latent Dirichlet Allocation (LDA) Blei et al. (2003) and Random Forest classifier to group tweets into topics for analysis. Recently, authors used a tensor factorization method to identify misinformation in COVID19 tweets and display their spatio-temporal distribution Balasubramaniam et al. (2021, 2020) and sentiment analysis Singh et al. (2021). Studies have also been published to analyse climate change-related tweets and understand what are the topics of discussion, how the tweet volume and sentiment changed over time Abdar et al. (2020); Ballestar et al. (2020); Dahal et al. (2019). Authors in Lansley and Longley (2016) applied topic modelling on a corpus of geotagged tweets collected from the London sphere. Topic modelling has also been used to estimate the similarity between users in location-based social networks Lee et al. (2016) and to estimate the relatedness of businesses based on business descriptions Shi et al. (2016).

In this paper, we propose a Semi-supervised Neural Topic Model (SNTM) for topic analysis and an Informed Neural Network (INN) model for Sentiment Analysis. SNTM is inspired by LDA Blei et al. (2003); Gao et al. (2017) and implemented using a Variational Auto-Encoder (VAE) model Kingma and Welling (2013). INN integrates prior (expert) knowledge into the training process in addition to the training data. We rigorously evaluate these two models using several datasets. Then, we apply these two models for analysing a large Spatio-temporal tweet dataset of the Australian sphere Twitter^1^ containing certain keywords relating to COVID19. Additionally, we apply volume analysis, Dynamic Topic Modelling Blei and Lafferty (2006), and Semantic Brand Score (SBS) Fronzetti Colladon (2018) to obtain an insight into COVID19 outbreak in different states and cities of Australia over time.

The volume analysis aims to identify basic geospatial and temporal facts from the dataset which will facilitate subsequent analysis such as sentiment and topic into context. Topic modelling extracts topics present in the dataset and dynamic topic modelling shows how those topics evolve over time. Sentiment analysis determines the sentiment of every tweet to show how the community sentiments change over time. Impact analysis generate networks of concepts/words from the text collection and uses those networks to measure how differently the concepts/words impact a discussion. The analytical findings are then discussed and evaluated along with the comparison with independent observations such as government reported instances and news on newspapers.

To the best of our knowledge, this work is a first in-depth study of understanding Australian people’s perception of this ongoing COVID19 pandemic using a large Twitter data collection. More specifically, this study makes the following main contributions. (a) It proposes a Semi-supervised Neural Topic Model with high diversity and coherence for topic analysis. It then applies the model to understand what topics related to COVID19 have been discussed in communities. (b) It proposes a simple but accurate Informed Neural Network model for Sentiment Analysis. Further, it applies the INN model to understand the COVID19 related sentiments in communities over time. (c) It applies SBS in a unique setting to investigate the impact of COVID19 related concepts/words in social media discussion. (d) It investigates how closely the insights into the local outbreak match independently observed phenomena in space and time.

## The proposed experimental methodology

### Research objectives

**Fig. 1:**
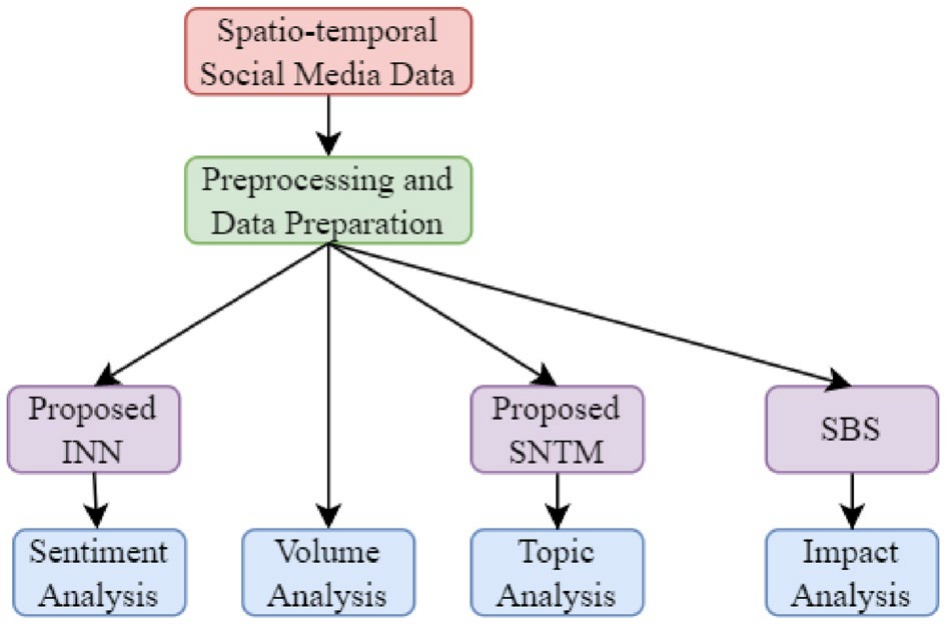
The experimental workflow

The aim of this study is to use social media analysis to uncover what is happening in communities and to give insight into (a) how the virus and lockdown is affecting community emotions, (b) understanding the main topics or themes emerging and evolving in the conversation, and (c) impact of different COVID19 related concepts. We conduct spatio-temporal analysis of volume, sentiment, topic, and impact to a large volume of COVID19 related tweets from the Australian sphere as shown in Fig. 1. We have collected a dataset of tweets from the Australian sphere containing geospatial and temporal values. The dataset is then preprocessed and prepared for volume, sentiment, topic, and impact analysis.

### Data collection and preparation

As we practice social distancing during COVID19 outbreaks, our embrace of social media becomes higher. Major social media platforms have emerged as critical information purveyors during the expanding pandemic. Twitter’s number of active users in the first three months of 2020 increased by 23% compared to the end of 2019, which is about 12 million more users. We collected two group of datasets: (a) Australian Sphere Dataset for getting insight into COVID-19 situation in Australia, and (b) Model Evaluation Datasets for evaluating proposed models.

#### Australian sphere dataset

We collected Twitter conversation in the Australian Sphere on COVID19 starting from 27 November 2019 when the first break out occurred in China. The data collection is done via the Queensland University of Technology (QUT) facility of Digital Observatory^2^ using the Twitter Stream Application Programming Interface (API). The dataset consists of 2.9 million tweets from 27 November 2019 to 7 April 2020. Every tweet in the dataset contains or uses as a hashtag at least one of the following keywords: coronavirus, covid19, covid-19, covid_19, coronovirusoutbreak, covid2019, covid, and coronaoutbreak.

The body of each tweet, i.e. tweet message, is used for analysing sentiment, topics and impact. Location and time information of each tweet gives it spatio-temporal dimensions. The location information for each tweet comes from either of three sources based on their availability: (a) tweet location, i.e. the location user was in when the tweet was posted; (b) user location, i.e. residence of the user; or (c) location mentioned in the tweet message. The locations are mapped to capital cities, states, or the country Australia depending on how granular level location information are extracted. The time information of each tweet comes from the time and day the tweet was posted. Table 1 shows a few examples.

For preprocessing, we removed stopwords, punctuations, and invalid characters. We dropped any non-English tweets. We fixed repeating characters, converted text to its lowercase, replaced an occurrence of link or URL with a token namely xurl, and stemmed the text. We used Named-entity Recognition (NER) from spacy^3^ to extract locations.

#### Model evaluation datasets

For evaluating the proposed sentiment analysis model, we collected the sentiment140 (Senti140) (Go et al. 2009), COVID19Senti (Sentiment Analysis 2021) and GeneralSenti (Twitter sentiment xxxx) datasets. Sentiment140 contains 1.6 million tweets annotated for two classes of sentiments: positive and negative. COVID19Senti contains around 41,000 tweets annotated with five types of sentiments that we grouped into two: positive and negative. GeneralSenti has around 32,000 annotated tweets that include two groups of sentiments: positive and negative.

For evaluating the proposed topic analysis model, we used three datasets: COVID19Senti (as discussed above), EastAsianHate (Vidgen et al. 2020); Bashar et al. 2021) and RandomHate (Twitter hate xxxx; Bashar et al. 2021). EastAsianHate contains around 8,000 tweets annotated as whether a tweet is East Asian relevant or not and, if so, what is the stance. RandomHate has around 4,000 random tweets annotated as whether a tweet is hate speech or not. We use the same preprocessing as the Australian Sphere data to prepare these datasets.

**Table 1:** Examples of tweets in the the Australian Sphere dataset. @someone and @something is used to anonymise a person or an organisation mentioned in the tweet, a token URL is used to replace any occurrence of hyperlink or URL. Location and time are extracted from the tweet metadata

Location	Tweet Text	Time
Australia	RT @someone: Coronavirus patient sealed in a PLASTIC TUBE to avoid contamination URLl @something	22/01/2020 17:47
Melbourne	RT @someone: Me seeing the doomsday clock going to a 100 seconds, Australia on fire and the coronavirus all trending on the same day \n #AustraliaOnFire\n #CoronavirusOutbreak\n #DoomsdayClock\n #Wuhan\n #coronovirus URL	24/01/2020 0:50
Tasmania	RT @someone: BREAKING: virologist who helped identify SARS says a bigger #CoronavirusOutbreak is certain, conservatively estimating it could be 10x bigger than SARS because SARS was transmitted by only a few super spreaders in a more defined part of #China.\n URL	24/01/2020 13:26

### Volume analysis

Analysing the volume of tweets posted from a particular area at a particular time is an important step of data exploration that can provide interesting insights into observations Dahal et al. (2019). We analyse the number of tweets posted in each state and capital of Australia over time.

### Sentiment analysis

Sentiment analysis is used to identify the emotional state or opinion polarity in the samples. We propose an Informed Neural Network (INN) model to identify the sentiment of each tweet. We then aggregate tweets by location and time to obtain spatio-temporal distribution of sentiments. The following section gives a summary of the proposed INN architecture for sentiment classification.

#### Informed neural network for sentiment analysis

**Fig. 2:**
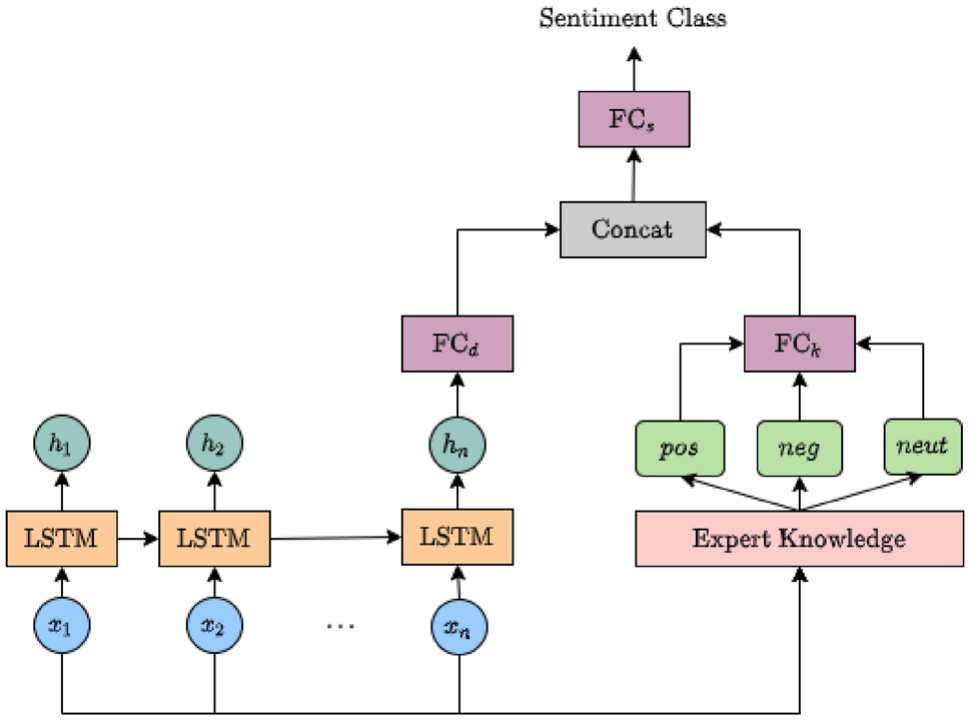
The proposed informed neural network (INN) architecture for sentiment analysis

**Fig. 3:**
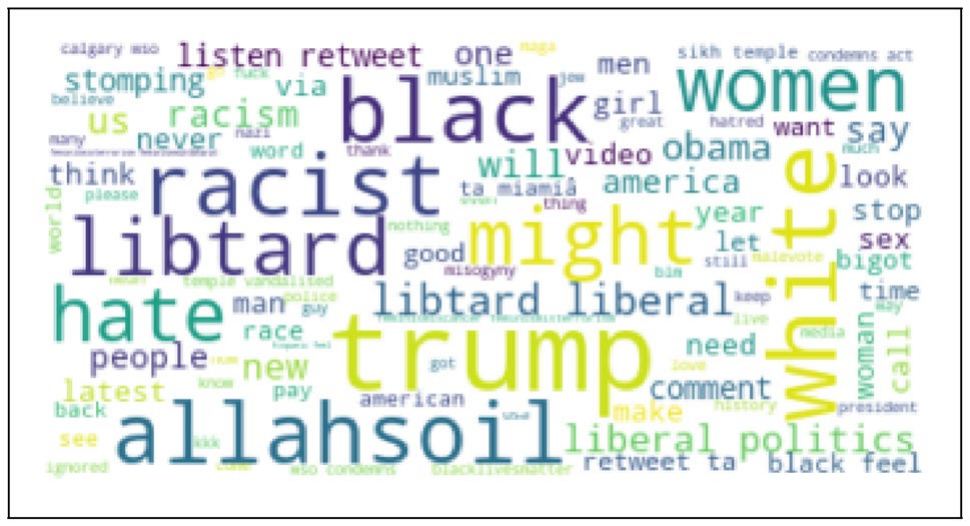
Word cloud obtained from the random hate data (Before COVID-19)

**Fig. 4:**
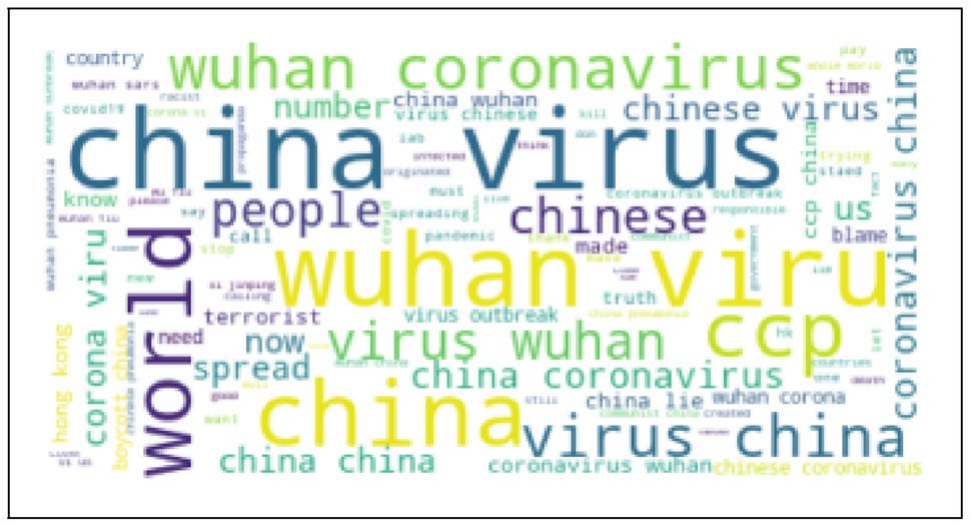
Word cloud obtained from the East Asian hate data (During COVID-19)

If there is not sufficient training data, especially when application domain is impacted by data shift, machine learning models can overfit and provide poor accuracy Bashar et al. (2021). During a suddenly changed situation such as COVID19 pandemic, domain is impacted by data shift as the underlying data distribution on twitter before pandemic and during pandemic changed significantly Bashar et al. (2021). Word clouds generated from two such datasets shown in Figs. 3 and 4 highlight the variations in two distributions, e.g. vocabulary, term frequency and the use of language. This problem can be potentially addressed by integrating prior knowledge into the training process von Rueden et al. (2019); Bashar et al. (2021). This process of integration is known as Informed Machine Learning von Rueden et al. (2019). Improving machine learning models by integrating additional prior knowledge into the training process has gained a huge research interest recently. Combination of data- and knowledge-driven approaches is becoming popular in many research areas von Rueden et al. (2019).

In essence, an informed machine learning model uses a hybrid information source that consists of data and prior knowledge. The prior knowledge is pre-existent and independent of learning algorithms. In this research, we propose an INN model for sentiment analysis that uses the sentiment as prior knowledge in the form of lexicon and rules obtained from VADER C. J. Hutto (2014). VADER lexicon consists of 7,500 features and has sophisticated linguistic rules to produce sentiment scores. The prior knowledge is utilised to estimate three sentiment scores (positive, negative, and neutral) for a given tweet (i.e. a candidate for analysis). These three scores reveal probabilistic relations between a text and sentiment and are used in regularising the INN model in the training process.

Figure 2 shows the architecture of the proposed INN Model for sentiment analysis. This architecture has two fully connected layers as two main components. One layer FC$_d$ learns the latent representation of the input data and another layer FC$_k$ learns the relevant prior knowledge representation of the input data. Firstly, each feature $x_i$, where $1 \le i\le n$, of a tweet of *n* words is sequentially fed to a LSTM unit for sequentially representing the features in the hidden states (or latent space) $h_i$. We take the final hidden state $h_n$ that accumulates all prior hidden states in the sequence as a summary and feed it to a fully connected layer FC$_d$. The output of this FC$_d$ is the latent representation of data in our model. The goal of this component is to learn sentiment prediction features from training data. However, if there are not enough training data, this component may overfit the training data. Therefore, simultaneously, we send the input features ($x_1, \dots x_n$) to Expert Knowledge component to obtain three scores (positive, negative and neutral) for a tweet based on prior knowledge. These three scores are fed to a fully connected layer FC$_k$. The output of this FC$_k$ is the prior knowledge representation of sentiment analysis. The goal of this component is to regularise sentiment prediction in final layer $FC_s$. This regularisation helps in reducing model overfitting when there are not enough labelled data. The outputs of FC$_d$ and FC$_k$ are concatenated [FC$_d$, FC$_k$] and fed to final fully connected layer FC$_s$. Output of FC$_s$ is distributed over sentiment classes and normalised through a softmax function. Our proposed model INN is evaluated in Sect. 3.2.

### Topic analysis

A variety of subjects or topics are usually discussed in the tweets over time. Knowing those topics and how they evolve is important to understand the dynamics of discussion related to COVID19. Because of the large size of the tweet dataset, it is very difficult, if not impossible, to read all of the tweets for finding out their topics. Topic models are the most popular statistical methods that analyse the words in a document collection to discover the themes that run through the data collection Blei and Lafferty (2006); Blei et al. (2003). This analysis reveals how those themes are connected and how they change over time. We propose a Semi-supervised Neural Topic Model (SNTM) to discover topics. The following section gives a summary of the proposed SNTM architecture.

**Fig. 5:**
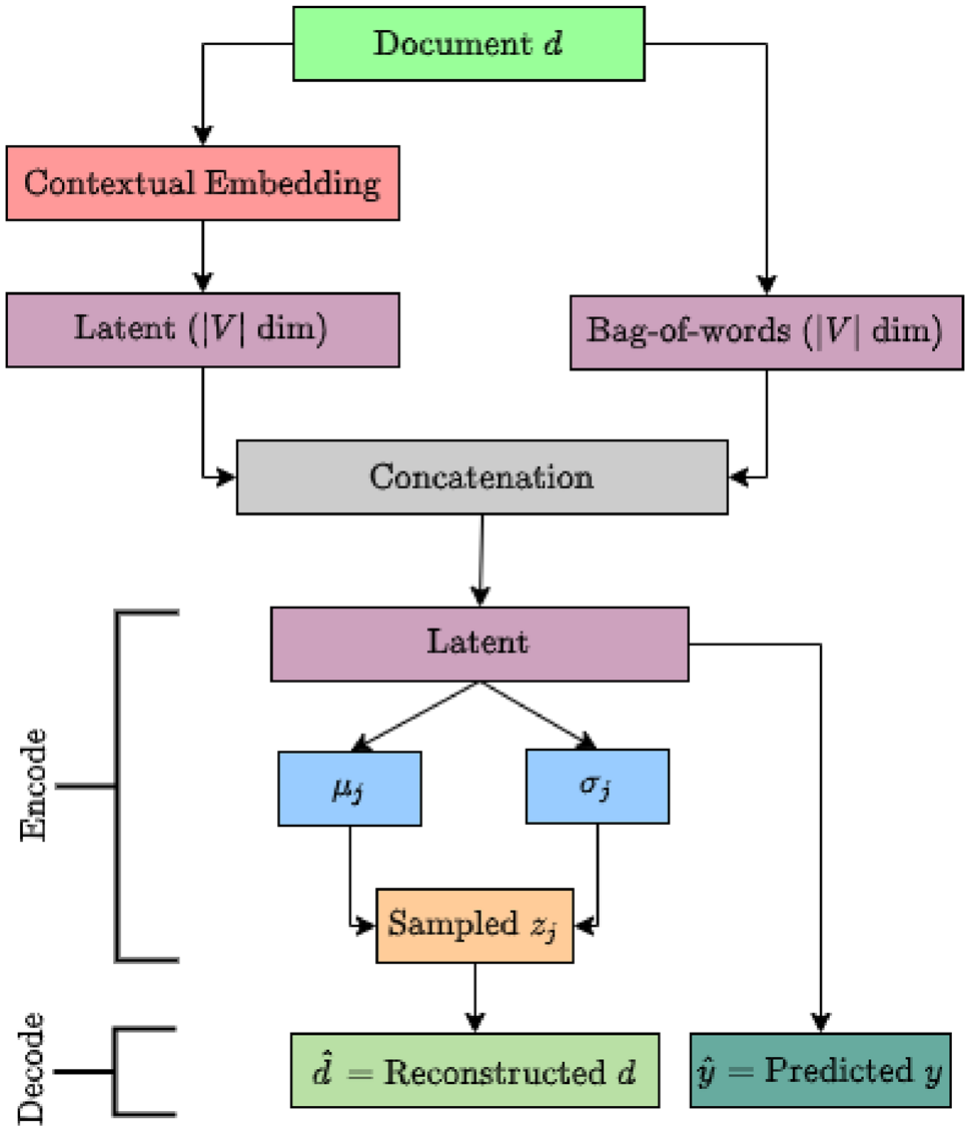
Semi-supervised neural topic model (SNTM)

#### SNTM architecture

The proposed SNTM, shown in Fig. 5, is inspired by LDA Blei et al. (2003) and implemented utilising a neural network architecture named Variational Auto-Encoder (VAE) Kingma and Welling (2013).

The idea behind LDA Blei et al. (2003) is that observed terms in each document (or text such as tweet) are generated by a document-specific mixture of corpus-wide hidden topics Blei et al. (2003); Bashar and Li (2017); Alharbi et al. (2018). It assumes the number of hidden topics are fixed to *T*. It represents a topic $z_j$ as a multinomial probability distribution over the vocabulary (i.e. *V* terms) as $p(t_i|z_j)$, where $1 \le j \le T$ and $\sum _i^V p(t_i|z_j) = 1$. LDA represents each document *d* as probabilistic mixture of topics as $p(z_j|d)$. Therefore, the probability distribution of *i*th term in a document *d* can be modelled as a mixture over topics: $p(t_i|d) = \sum _{j=1}^T p(t_i|z_j)p(z_j|d)$. Here the only observable variable is $p(t_i|d)$. The other two variables $p(t_i|z_j)$ and $p(z_j|d)$ are hidden. In this research, we use VAE Kingma and Welling (2013) for learning the two hidden variables $p(t_i|z_j)$ and $p(z_j|d)$. Using VAE, we directly map a document to an approximate posterior distribution $p(t_i|d)$. More specifically, VAE maps *d* to $z_j$ for $1 \le j \le T$ using the encoder part, i.e. encoder part approximates $p(z_j|d)$. Then, decoder part maps $z_j$ to $t_i$ for reconstructing the document *d*, i.e. decoder part approximates $p(t_i|z_j)$. Overall the network approximates $p(t_i|d) = \sum _{j=1}^T p(t_i|z_j)p(z_j|d)$. Suppose the reconstructed document is ${\hat{d}} = p(t_i|d)$ and $z_j$ is sampled from ${\mathcal {N}}(\mu _j, \sigma _j)$, i.e. $z_j \sim {\mathcal {N}}(\mu _j, \sigma _j)$. The loss $L_1$ for the reconstruction is calculated as follows.

$$\begin{aligned} L_1 = \Vert d - {\hat{d}} \Vert ^2\end{aligned}$$

VAE regularises distribution ${\mathcal {N}}(\mu _j, \sigma _j)$ by enforcing the distributions to be close to a standard normal distribution ${\mathcal {N}}(0,1)$ through minimising KL divergence Kullback and Leibler (1951) of them. This regularisation prevents the model to encode data far apart in the latent space and encourage returned distributions to overlap. Thereby, this model satisfies expected continuity and completeness, where continuity means two close points in the latent space should not yield completely different contents once decoded and completeness means for a chosen distribution, a point sampled from the latent space should yield meaningful content once decoded. Therefore, the loss $L_2$ for the reconstruction is calculated as follows.

$$\begin{aligned} L_2 = \Vert d - {\hat{d}} \Vert ^2 + KL[{\mathcal {N}}(\mu _j, \sigma _j), {\mathcal {N}}(0,1)] \end{aligned}$$

However, random sampling (to obtain $z_j$) that occurs in the encoder part of VAE will prevent backpropagation through the network. Therefore, the sampling process is expressed using reparametrisation trick that allows the gradient descent possible Kingma and Welling (2013). Reparametrisation is done as $z_j = \mu _j + \sigma _j \zeta$, where $\zeta = {\mathcal {N}}(0,1)$.

In some cases, we may have a portion of documents that are already labelled to certain topics or classes. Such labels can be leveraged to learn discriminative features useful for gathering similar features under similar topics, and separate topics from each other. Therefore, we use a classification layer in decoder part of the model. The input to this layer comes from the latent representation of document in encoder part of VAE. Suppose the label of a document is *y* and the predicted label is ${\hat{y}}$. The overall loss of our deep topic model can be written as.

1 $$\begin{aligned} L = | y - {\hat{y}}| + \Vert d - {\hat{d}} \Vert ^2 + KL[{\mathcal {N}}(\mu _j, \sigma _j), {\mathcal {N}}(0,1)] \end{aligned}$$

To improve topic coherence, similar to ProdLDA Srivastava and Sutton (2017), we assume distribution over individual words is a product of experts Srivastava and Sutton (2017) rather than the mixture model used in LDA. Similar to Combined Topic Model (CTM) Bianchi et al. (2020), we use contextualised document embeddings from Sentence-BERT (a extension of BERT that allows quick generation of sentence embeddings) Reimers and Gurevych (2019). Firstly, the document embeddings are projected to the same dimensionality as the vocabulary size through a latent layer, and then concatenated with the Bag-of-words representation. Our proposed model SNTM is evaluated in Sect. 3.3.

#### Topic processing

Choosing a reasonable number of topics is important. Too few topics could lead to merging distinct topics whereas too many topics could result in fragmented topics that otherwise could make a cohesive topic together. We manually evaluate topic models with topics ranging from 5 to 50, to determine the optimal number of topics.

In Bag-of-words representation, we remove keywords and hashtags (e.g. covid19, coronavirus, etc.) that we used for collecting our tweets. This ensures that the topics discovered are meaningful and not dominated by the same top words. We also remove rare words (i.e. words with a very low frequency) to reduce noise in the topics.

Finally, we use dynamic topic modelling in Blei and Lafferty (2006) to observe how topics evolve over time. Dynamic topic modelling can capture the evolution of topics in a sequentially organised collection of tweets or documents. For example, the tweets published in different time periods can be related to a specific topic namely *coronavirus cure*, however the topic of ‘coronavirus cure’ can appear differently in later time stage than the early stages. The themes in a tweet collection evolve. It is of interest to explicitly model the dynamics of the underlying topics. In our setting, tweets are grouped by weeks and ordered by successive weeks, to understand their weekly evolution during the period of data collection.

### Impact analysis

The Semantic Brand Score (SBS) is used in estimating the impact or importance of brands in a text collection in Business domains Fronzetti Colladon (2018). In this paper, we use SBS in a novel fashion to understand the impact of different COVID19 related concepts or entities via the social media discussions. SBS is measured based on graph theory that combines methods of social network and semantic analysis using the word co-occurrence network Fronzetti Colladon (2018). Same as in the original paper Fronzetti Colladon (2018), we calculate SBS as the standardised sum of three components: prevalence, diversity, and connectivity.

Prevalence *PREV*(*c*) calculates the number of times a word/concept *c* is mentioned in a tweet collection Fronzetti Colladon (2018). Prevalence is associated with the idea of brand awareness assuming that when a concept is frequently mentioned, its recognition and recall is increased. Diversity *DIV*(*c*) of a word/concept *c* estimates the heterogeneity of concepts surrounding it Fronzetti Colladon (2018). It is the degree of centrality in the co-occurrence network. The degree of centrality is estimated by counting the number of edges directly connected to the concept node *c*. Connectivity *CON*(*c*) of a word/concept *c* estimates its connectivity with respect to a general discourse Fronzetti Colladon (2018). It represents the ability of the concept node *c* to act as a bridge between other nodes in the network. Connectivity is widely used in social network analysis as a measure of influence or control of information that goes beyond direct links. It is estimated as

$$\begin{aligned} CON(c) = \sum _{j \ne k} \frac{d_{jk}(c)}{d_{jk}} \end{aligned}$$

where $d_{jk}$ is the number of the shortest paths linking any two nodes *j* and *k*, and $d_{jk}(c)$ is the number of those shortest paths that contain the given concept node *c*.

The Semantic Brand Score is estimated as Fronzetti Colladon (2018):

$$\begin{aligned}&SBS(c) = \frac{PREV(c)-{\overline{PREV}}}{std(PREV)} + \frac{DIV(c)-{\overline{DIV}}}{std(DIV)} \nonumber \\&+\frac{CON(c)-{\overline{CON}}}{std(CON)} \end{aligned}$$

where ($\overline{.}$) represents the mean value and *std* represents the standard deviation.

## Experimental results

This section presents the results and observations from volume analysis, topic modelling, sentiment detection, and semantic brand score estimation conducted on the Australian Sphere dataset to understand COVID19 pandemic outbreak. This dataset contains 2.9 million tweets from 27 November 2019 to 7 April 2020. The temporal dimension (27 November 2019 to 7 April 2020) of the tweet collection is discretized by weeks (roughly 17 weeks) or days as appropriate to the nature of the analysis. The geospatial dimension is discretized by Australian States and capital cities. The tweet user location that does not list city but lists the country (i.e. Australia) is categorised as Australia (au) in the following tables and graphs. The locations of a small portion of tweets, that could not be extracted or mapped to our selected categories, are categorised as others (oth).

### Volume analysis

**Fig. 6:**
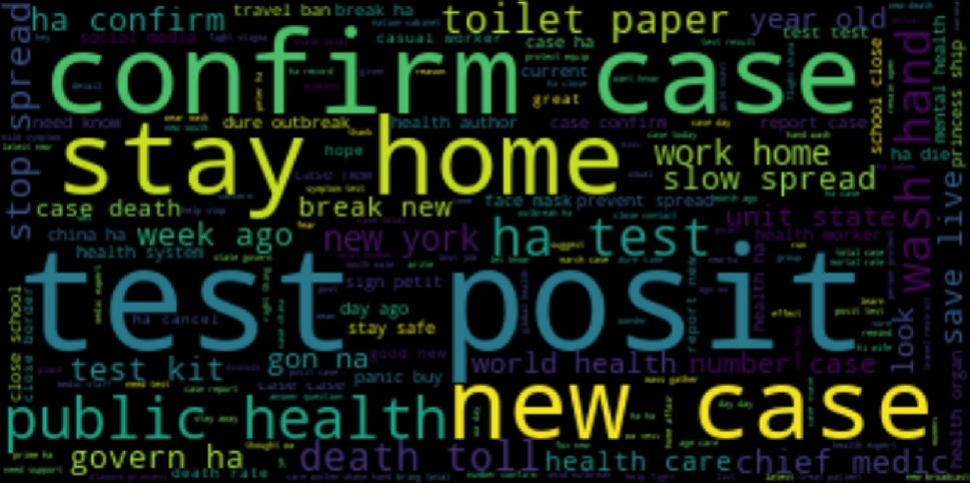
A word cloud generated from the Australian sphere tweet collection. The bigger and bolder a word appears, the more often it is mentioned in the collection

Figure 6 shows a word cloud generated from the entire tweet collection in the Australian Sphere dataset. It gives a quick look into the subjects Australian people discussed during this COVID19 pandemic period. Subjects such as ‘stay home’, ‘work from home’, ‘toilet paper crisis’ ‘slow the spread’, etc. are commonly discussed.

**Fig. 7:**
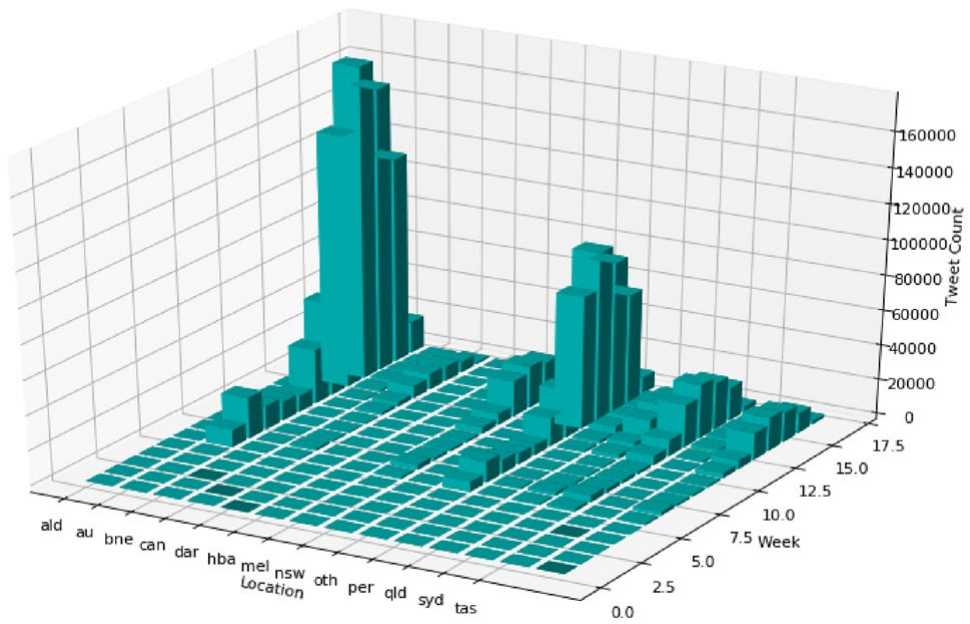
Geospatial and temporal distribution of tweet count in the Australian sphere tweet collection

**Fig. 8:**
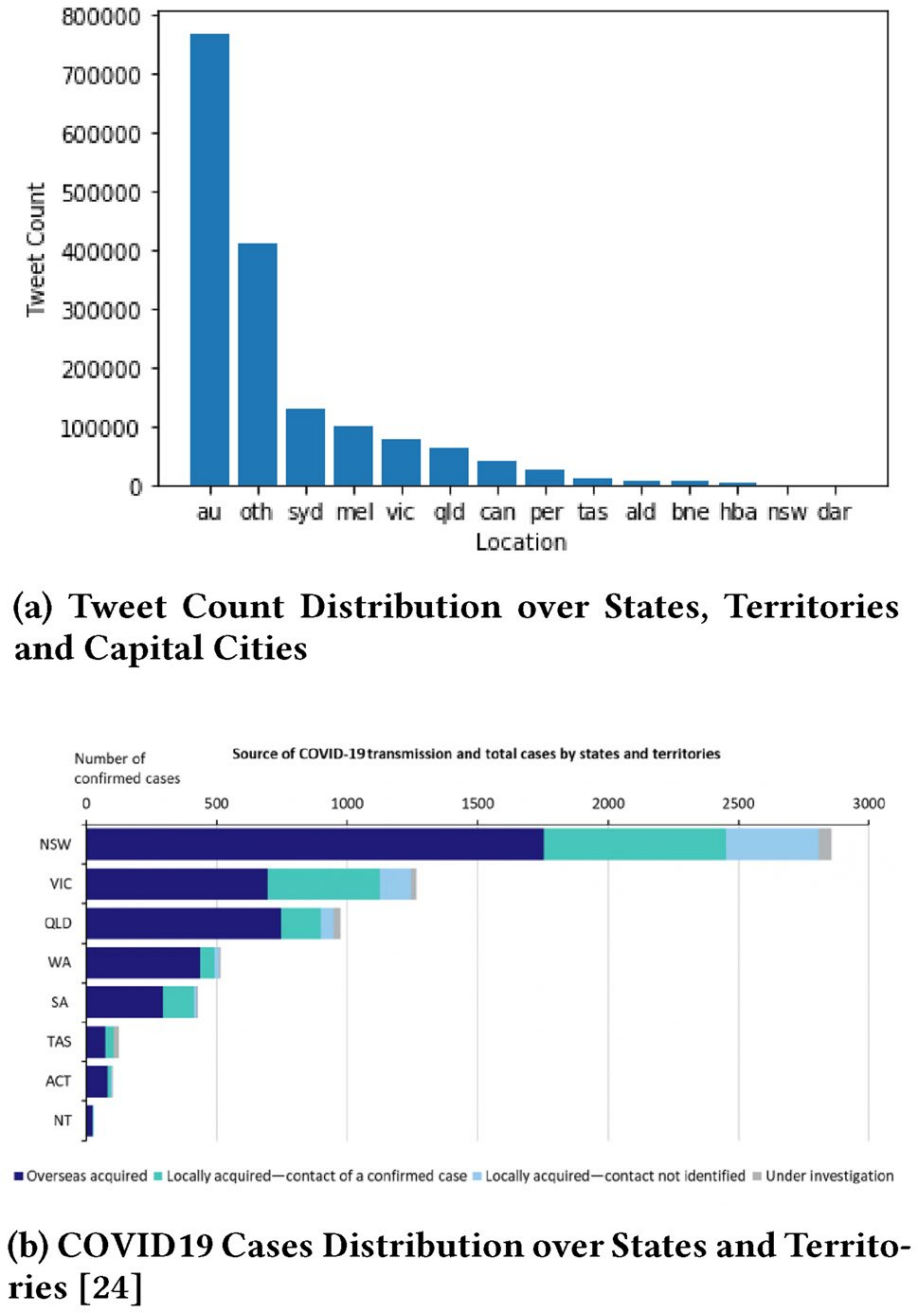
Correlation between *Tweet Counts* and *COVID19 Cases* Distributed over States, Territories and Capital Cities

**Fig. 9:**
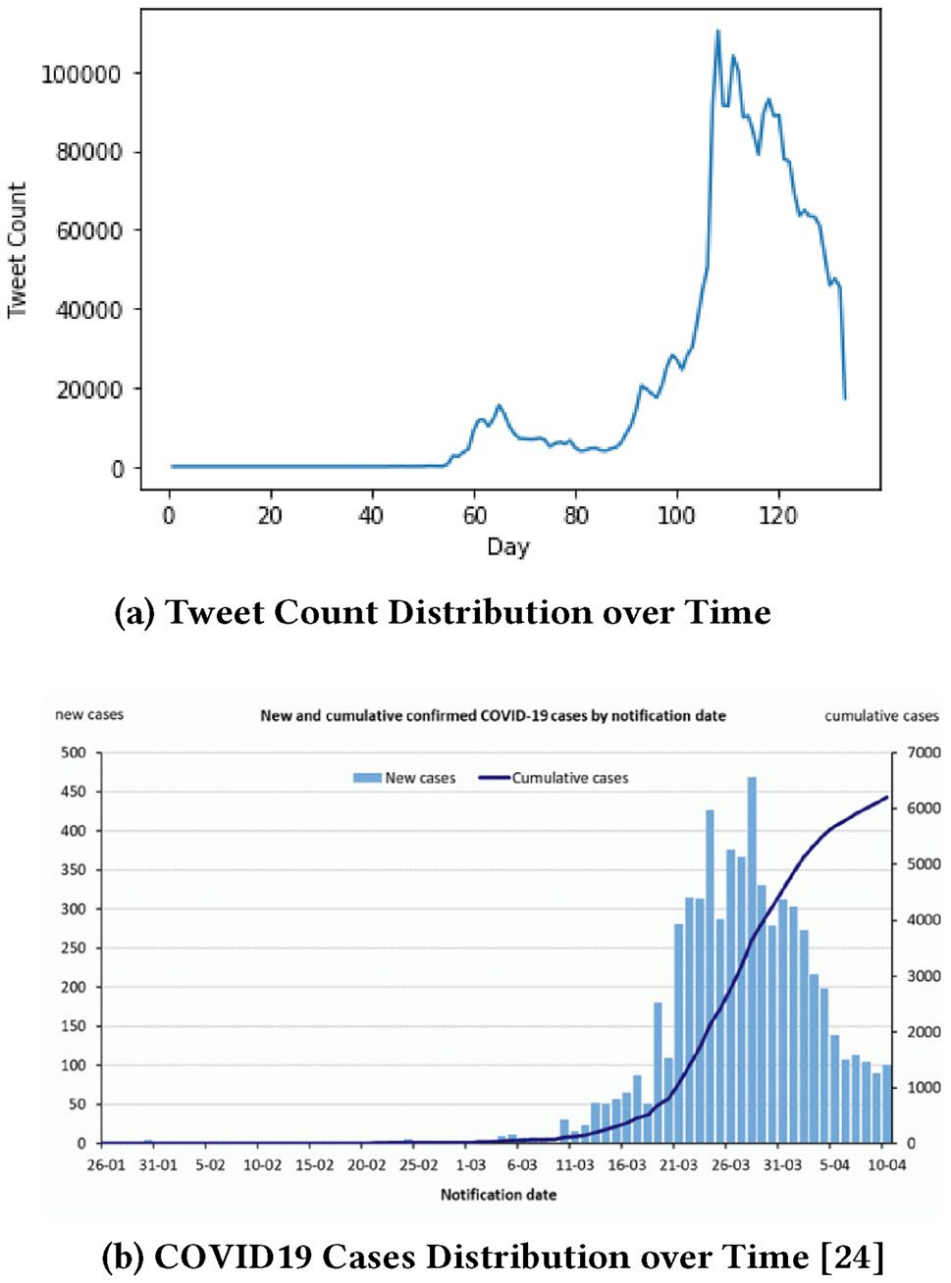
Correlation between *Tweet Counts* and *COVID19 Cases* Distributed over Time

Figure 7 shows the geospatial and temporal distribution of tweet counts in the collection. A significant change in tweet counts over locations and weeks can be noted throughout the time period. For a closer examination, we separate geospatial and temporal dimensions in Figs. 8 and 9, respectively.

Figure 8a shows the number of tweet counts in states, territories and capital cities of Australia. Figure 8b shows the actual number of COVID19 positive cases in states and territories of Australia. A strong correlation can be noted between tweet counts and COVID19 cases. The higher the number of COVID19 cases in a location, the higher is the number of tweets there. For example, the highest number of COVID19 related tweets were observed in Sydney (syd) (i.e. the capital city of New South Wales (nsw)), where the highest number of COVID19 cases occurred in nsw. The second and the third-highest number of COVID19 related tweets were observed in Melbourne (mel) (i.e. the capital city of Victoria (vic)) and vic, respectively, where the second-highest number of COVID19 cases occurred in VIC. The same is true for Queensland (qld). Other cities follow a similar pattern with minor order variations.

Figure 9 shows the correlation between tweet counts and COVID19 cases distributed over time. A comparison between Figs. 8a and 9b shows that the number of COVID19 related tweets over time is strongly correlated with the number of new COVID19 positive cases by the notification date.

Figure 8a shows that when COVID19 hit China on 27 November 2019, there were not many discussions held in Australian space. A noticeable number of coronavirus related tweets started to be posted after 60 days or around eight weeks, i.e. end of January. Next one week the number increased and then started to fall. The main burst of tweets started after another 30 days or 4 weeks, i.e. the end of February. This might be because this time several people in Australia from overseas were identified COVID19 positive. The number exponentially increased for the next 20 days and reached its peak by the third quarter of March. This exponential increase might have occurred because during this time many Australian were identified COVID19 positive and some of them were reported dead. Then it started to fall gradually. This might be because during this time government introduced many policies and strict social distancing worked and the COVID19 infection death rate started to decrease.

### Sentiment analysis

Firstly, we present the comparative performance of the proposed INN model with the state-of-the-art deep learning models to show its better performance. Once established, we then show the useful findings gained by the INN model on the Australia Sphere data to understand how the virus and lockdown are affecting the community’s emotions.

#### Comparative performance of the proposed INN model

Table 2 compares the experimental performance of the proposed INN model with baseline models Vanilla LSTM (VLSTM) Hochreiter and Schmidhuber (1997) and Universal Language Model Fine-tuning (ULMFiT) Howard and Ruder (2018). We use the same LSTM architecture in INN and VLSTM to represent the data. INN has an additional neural network part to represent the prior knowledge that VLSTM does not have. ULMFiT is a state-of-the-art classification model that has a medium size parameter set. Compared with INN and VLSTM, ULMFiT is a large neural network based on LSTM and uses a pretrined language model. For evaluating models, we used six metrics such as Accuracy, Precision, Recall, F$_1$ measure, Cohen Kappa Score, Area Under Curve. A description of these metrics is available in Bashar et al. (2020).

Table 2 shows that, in all six measure, INN performs significantly better than VLSTM and ULMFiT on the COVID19Senti dataset. Compared with VLSTM, the performance improvement of INN is 27.79% for Cohen Kappa Score, 11.61% for Area Under Curve, 9.63% for Accuracy, 9.43% for Precision, 6.98% for F$_1$ Measure and 4.52% for Recall. Compared with ULMFiT, the performance improvement of INN in different measures range from 8.44% to 2.3%. On the GeneralSenti dataset, the performance of INN is better than VLSTM in all six measures. For example, the performance improvement is 5.51% for Recall, 4.40% for F$_1$ Measure and 4.76% for Cohen Kappa Score. On this dataset, INN outperforms ULMFiT in all the measures except precision. Even though ULMFiT has marginally better precision, its Recall and F$_1$ measure is lower. In other words, ULMFiT achieves better precision by sacrificing Recall, i.e. ULMFiT misses more number of positive sentiments than INN. On the Senti140 dataset, INN performs marginally better or similar to VLSTM and ULMFiT.

Senti140 is a large dataset that include 1.6 M annotated tweets. Due to its large size, this dataset covers sufficient information. Consequently, the addition of prior knowledge in INN does not contribute much for performance enhancement. On the other hand, COVID19Senti and GeneralSenti are small datasets where integration of prior knowledge contributed the most. Even if the prior knowledge has limited contribution in challenging dataset (such as GeneralSenti) due to context dependency, prior knowledge can still help in improving the overall model such as INN. For example, when we used prior knowledge-based Lexical sentiment analysis on GeneralSenti dataset, we obtained Accuracy 0.219607161, Precision 0.04735092, Recall 0.526367669, F1 Measure 0.086885779, Cohen Kappa Score -0.048870461 and Area Under Curve 0.361347359. In spite of this poor performance from Lexical sentiment analysis, prior knowledge improved the performance of INN in this dataset.

The significance of this finding is that prior knowledge integration with machine learning model can significantly benefit small datasets and such integration is always beneficial, even for large datasets. In this research, we only utilised lexicon and rules C. J. Hutto (2014) as prior knowledge. Exploring other source of prior knowledge will prove the robustness of the proposed model INN in other tasks. We will investigate this in our future work.

**Table 2:** Comparing experimental results of INN with VLSTM and ULMFiT

	COVID19Senti			GeneralSenti			Senti140		
	INN	VLSTM	ULMFiT	INN	VLSTM	ULMFiT	INN	VLSTM	ULMFiT
Accuracy	**0.918**	0.837	0.888	**0.966**	0.962	**0.966**	**0.801**	0.797	0.797
Precision	**0.934**	0.854	0.913	0.812	0.789	**0.841**	**0.807**	0.797	0.786
Recall	**0.935**	0.895	0.908	**0.629**	0.596	0.596	0.794	0.798	**0.815**
F1 Measure	**0.935**	0.874	0.910	**0.709**	0.679	0.698	**0.800**	0.797	**0.800**
Cohen kappa score	**0.823**	0.644	0.759	**0.691**	0.660	0.680	**0.603**	0.594	0.593
Area under curve	**0.911**	0.817	0.880	**0.809**	0.792	0.794	**0.801**	0.797	0.797

Boldfaced numbers indicate best values.

#### Sentiment analysis finding on the Australian sphere dataset

**Fig. 10:**
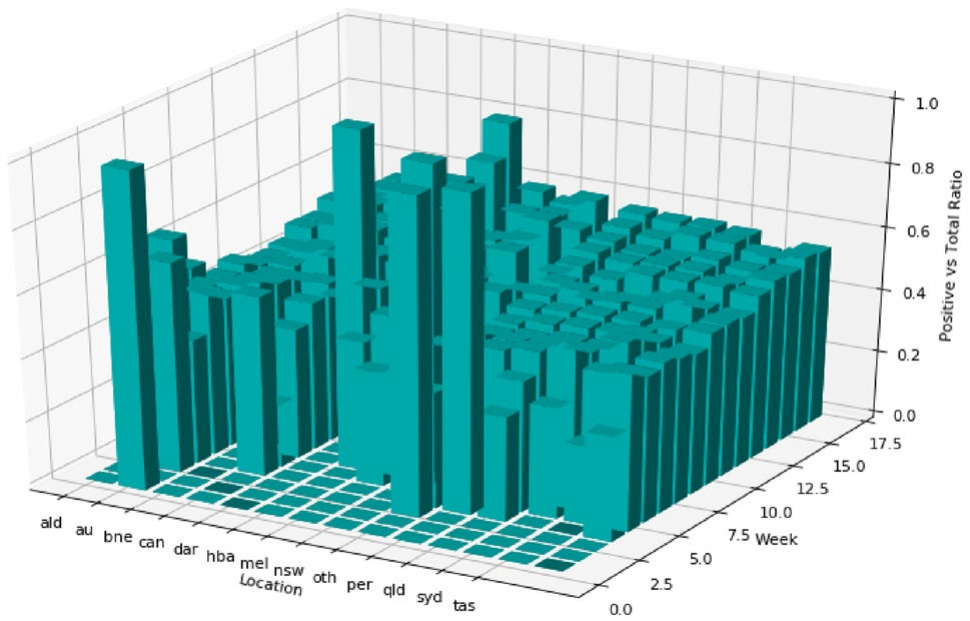
Geospatial and temporal distribution number of positive vs total tweet ratio

The following results of sentiment analysis are based on the proposed INN-based model applied on Twitter conversation in the Australian Sphere dataset. Figure 10 shows geospatial and temporal distribution of the ratio of positive sentiment tweet counts vs total tweet counts. As soon as COVID19 hit the world, the positive sentiments dropped sharply (roughly from 85% to 48% on average). The percentage stayed there for up to around 12 weeks. Then it gradually changed for three weeks with a very marginal positive increment. For the final two weeks, the increment was a bit more than the previous three weeks.

A possible explanation of the trend can be given as follows. As soon as COVID19 hit the world, the online community got shocked by the news. It took some time for world leaders to come up with plans on how to combat COVID19. During this period (12 weeks) people remained stressed. When the world leaders explained their combat plans and ideas, twitter users talked about those positive initiatives during this period (three weeks). In the final two weeks, the Australian government announced social safety plans, e.g. economic aids to organisations, businesses, and individuals; it announced more strict rules for social distancing and the COVID19 infection curve started flattening. People started to become slightly comfortable and discussed these positive aspects in their tweets. Consequently, the number of positive tweets increased. All these patterns show that monitoring conversational dynamics on social media can reveal how people feel during the COVID19 pandemic, and what initiatives work and makes people comfortable.

**Fig. 11:**
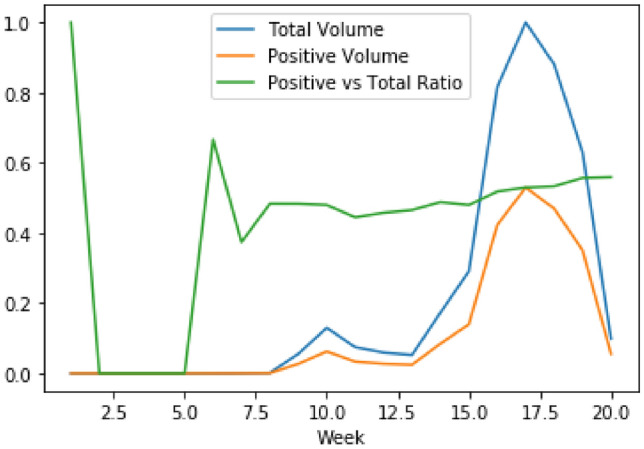
Temporal distribution of positive and total volume of tweets

**Fig. 12:**
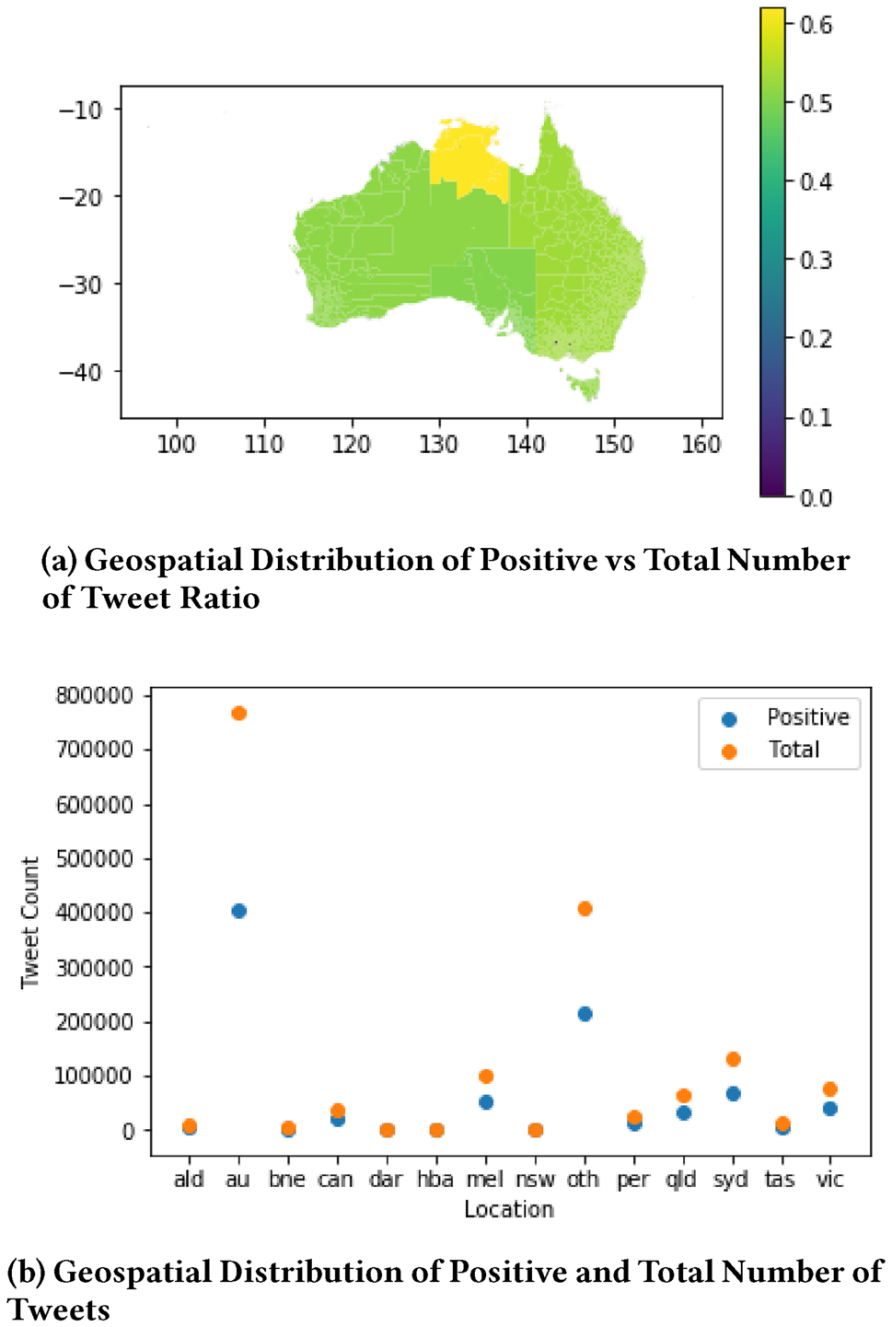
Geospatial distribution of sentiment

To have a closer look into the trend, the temporal and geospatial dimensions are decoupled in Figs. 11 and 12, respectively. Figure 11 shows the volume of COVID19 related tweets (total volume), the volume of positive tweets related to COVID19 (positive volume), and their ratio (positive vs total ratio). This figure shows that, roughly at any time, among all the COVID19 related posts, only 50% of them were positive. We see two significant drops in the ratio of positive sentiments, one is at the beginning when the world was hit by COVID19 and the next one is by week seven or third quarter of January 2020. During this period there were not many discussions of COVID19 in Australia. However, the second drop triggered an increase in the number of COVID19 related posts. In other words, this second drop alerted the community about the upcoming danger of COVID19. We can assume that the small number of tweets related to COVID19 might come from the people who are Journalists, social workers, health care workers, or people who are conscious of health issues.

During the period when a noticeable number of posts were related to COVID19 (week 8 to 18), there are two small drops in the ratio of positive sentiments. One in week 10 and another in week 14. Both drops are followed by a significant increase in the number of COVID19 related posts. Even though these two drops are small in sentiment ratio, the drops in the number of positive tweets were large enough to initiate triggers. It ascertains that monitoring the positive sentiment tweets can signal us the trigger in the increase in COVID19 related posts.

Figure 12a shows how the ratio of the number of positive tweets vs total tweets varies in Australian states and territories. It shows that all states and territories have the positive sentiment tweet ratio of around 0.5 except Northern Territory that has a slightly better ratio. This implies there is emotional stress in people over all the states and territories. However, this figure does not clearly capture the positive sentiment drop as cities are averaged over in the states and territories. In reality, some cities are affected more than others by COVID19. Therefore, we add capital cities in Fig. 9 along with states and territories.

Figure 12b shows the counts of COVID19 related tweets and positives tweets in states, territories, and capital cities. Capital cities and states that have a significant drop in positive tweet count are Sydney (syd), Melbourne (mel), Victoria (vic), and Queensland (qld). A comparison between Figs. 12b and 9b shows that these locations had most of the COVID19 cases. A drop in positive sentiment is correlated with the number of COVID19 cases. A drop in positive sentiment is also correlated with early mental health issues, informing that the community might need an allocation of mental health care resources in the near future.

Two interesting facts in Fig. 12b can be observed in varied behaviour between two pairs of state and its capital city, (qld, bne) and (nsw, syd) pairs. There is a significant drop in positive tweets in qld but not in bne. The majority of COVID19 cases in Queensland was in Gold Coast and other surrounding areas rather than Brisbane. Again, there is a significant drop in positive tweet count in syd but not in nsw. The majority of COVID19 cases was in Sydney rather than the other parts of nsw. This again emphasises that a drop in positive sentiment is directly correlated with the number of COVID19 cases.

### Topic analysis

Firstly, we present the comparative performance of the proposed SNTM model with the state-of-the-art topic modelling approaches to show its better performance. Once established, we show the useful findings gained by topic modelling on the Australia Sphere data to understand the main topics or themes emerging and evolving in the conversation.

#### Comparative performance of the proposed SNTM model

Table 3 compares the performance of the proposed SNTM model with three baseline models namely CTM Bianchi et al. (2020), LDA Blei et al. (2003) and Non-negative Matrix Factorisation (NMF) Andrzej CICHOCKI (2009). We evaluate the models using three metrics namely Normalised Pointwise Mutual Information (NPMI) Lau et al. (2014), External Word Embeddings Topic Coherence (EWETC) Ding et al. (2018) and Inversed Rank-Biased Overlap (IRBO) William Webber (2010). NPMI measures how related the top-*n* words of a topic are and take average over the *T* topics. It considers the words’ frequency in the original dataset. EWETC measures how similar the top-*n* words in a topic when their external word embeddings Mikolov et al. (2013) are considered. More specifically, it computes the average pairwise cosine similarity of the word embeddings of the top-*n* words in a topic, then it takes average over the *T* topics. In this setting, word embeddings from Mikolov et al. (2013) are used. IRBO evaluates how diverse the topics generated by a model are. It uses the reciprocal of the standard RBO William Webber (2010).

Table 3 shows that our proposed SNTM model performs the best overall. It always gives the best IRBO performance with a high score, which means the topics generated by SNTM are diverse. This might be because SNTM utilises contextual information and some label information. Since both SNTM and CTM use contextual information, their IRBO score is significantly better than LDA and NMF. As SNTM additionally utilises labelled information its IRBO score is better than the state-of-the-art-model CTM. EWETC score gives an estimation of coherence in a topic. For EastAsianHate and RandomHate datasets, SNTM gives the best EWETC score and this score is similar to NMF for COVID19Senti. NMF provides the best NPMI score for EastAsianHate and RandomHate datasets, while a similar NPMI score is achieved by SNTM and CTM for COVID19Senti. The reason for NMF providing the best NPMI score might be related to two facts. 1) both NMF and NPMI disregard the contextual information. 2) NPMI is computed on the original data as computing NPMI on an external corpus is expensive Bianchi et al. (2020). Ding et al. Ding et al. (2018) pointed out that topic coherence computed on the original data is inherently limited. Overall, SNTM gives superior results for topic modelling even though there are room for optimising the architecture of SNTM in terms of the number of hidden layers used and the number of neurons used in each layer. In our future work, we will investigate in that direction.

**Table 3:** Performance of the proposed SNTM model compared with other approaches

	EastAsianHate			RandomHate			COVID19Senti		
	$\uparrow$ NPMI	$\uparrow$ EWETC	$\uparrow$ IRBO	$\uparrow$ NPMI	$\uparrow$ EWETC	$\uparrow$ IRBO	$\uparrow$ NPMI	$\uparrow$ EWETC	$\uparrow$ IRBO
SNTM	− 0.304	**0.436**	**0.992**	− 0.519	**0.424**	**1.000**	− 0.280	0.419	**0.998**
CTM	− 0.278	0.393	0.984	− 0.413	0.326	**1.000**	**− 0.243**	0.384	0.996
LDA	0.008	0.365	0.638	− 0.021	0.393	0.897	− 1.645	0.346	0.646
NMF	**0.033**	0.355	0.760	**− 0.020**	0.419	0.942	− 2.592	**0.432**	0.850

Boldfaced numbers indicate best values.

#### Topic analysis finding on the Australian sphere dataset

**Fig. 13:**
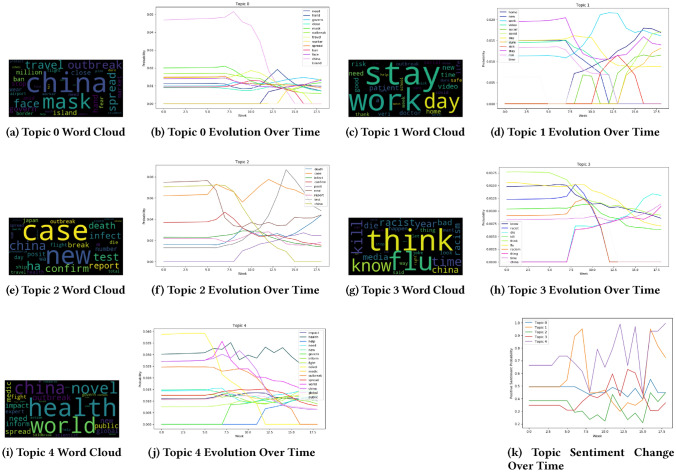
Topic clouds and topic evolutions

We now show some of the experimental results on how COVID19 related topics changed over time semantically, morphologically, and sentimentally in the Australian Sphere dataset using the results of SNTM. Figure 13 shows the evolution of five topics; Topic 0: controlling the spread, Topic 1: staying in isolation and working from home, Topic 2: COVID19 cases, Topic 3: racism against the Chinese community, and Topic 4: impact of COVID19 outbreak worldwide.

Topics 0, 2, and 4 show a similar trend even though their magnitude and change rate are different. A close investigation shows that these three topics share a high similarity in subject matter. On the other hand, Topics 1 and 3 do not resemble any trend. However, they somewhat inversely follow each other. It is apparent that all the topics evolved over time in terms of semantics, morphology and sentiment. For example, in Topic 0 that talks about controlling the spread of coronavirus, the words ‘need’ and ‘worker’ newly emerged during weeks 11 and 13, whereas the words ‘island’, ‘china’, ‘travel’, and ‘ban’ lost their significance during the weeks 12, 15, 16, and 17, respectively.

### Impact analysis

**Fig. 14:**
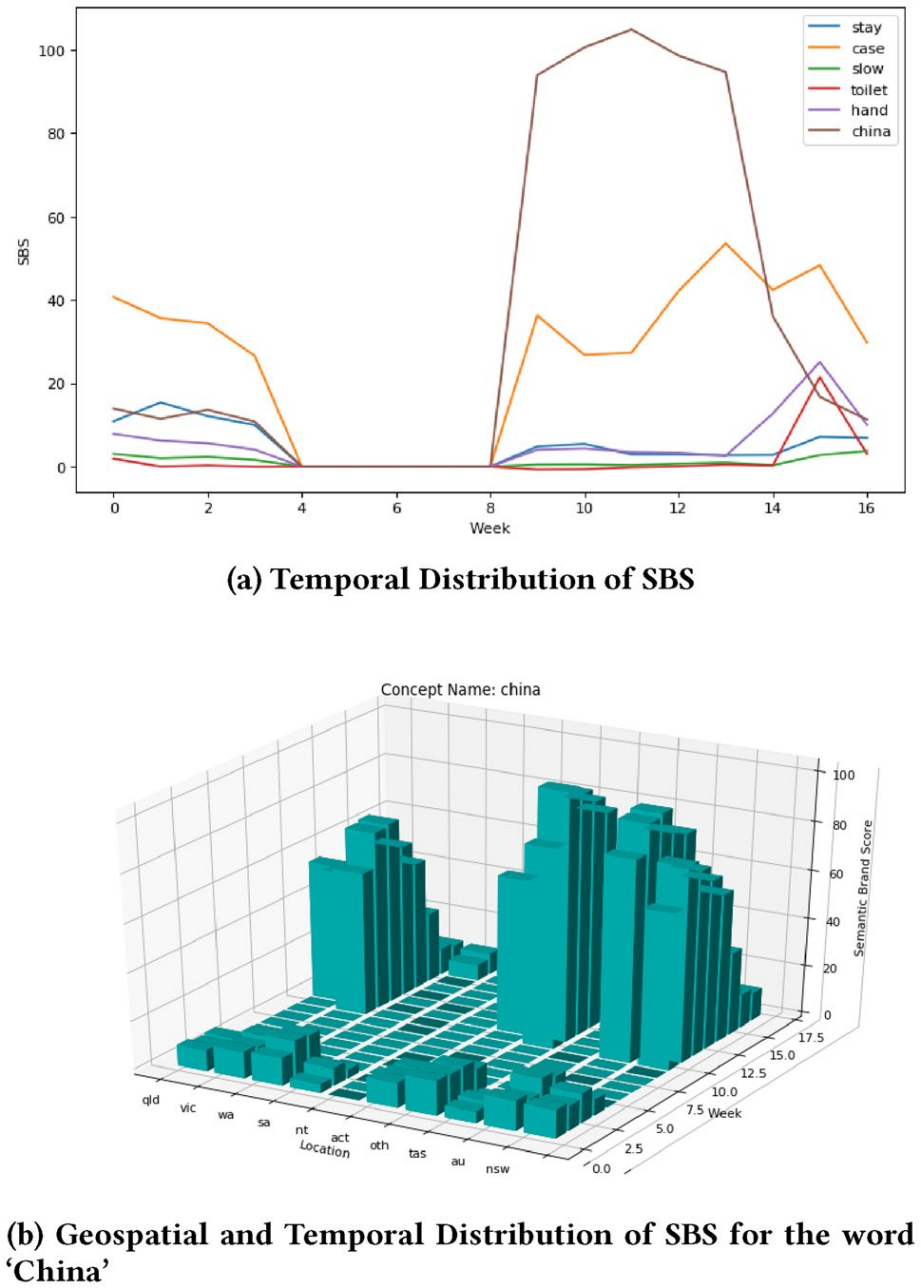
Distribution of SBS (Impact)

Semantic Brand Score (SBS) can capture the impact of concepts or words in text collections that might be useful for monitoring social matters or instances. Our topic analysis shows that some social instances during the COVID19 pandemic were ‘stay home’, ‘positive cases’, ‘slow the spread’, ‘wash your hands’, ‘toilet paper’, and ‘China’. We use SBS to get further insight into these instances by tracing SBS over time for keywords in those instances. Figure 14a shows the change of SBS over time for some of the words on these instances. This figure shows that china had the highest SBS score most of the time when compared with other words. The second highest SBS is counted for the word ‘case’ (i.e. positive cases). This reveals people’s interest on COVID19 positive cases and their implications on health, economy and jobs. The word ‘hand’ (i.e. wash your hands) had a stable SBS score during this period except for a spike in week 15. The word ‘toilet’ (i.e. toilet paper) had a low SBS with a spike in week 15 when some toilet paper related instances occurred in Australia (e.g. toilet paper sold out in most of the stores, people fighting over buying toilet papers, etc.).

Figure 14b shows how the SBS score varied in space and time for the word ‘China’. In a certain period and some places the word ‘China’ had a high SBS in COVID19 related tweets. This means, ‘China’ was mentioned in a lot of tweets, in a variety of topics, and a lot of topic of discussion involved the word ‘China’. This indicates that many diverse topics were influenced by the word ‘China’; and many topics were discussed in relation to the word ‘China’. This might have been influenced by the wrong assumption that China is responsible for the spreading of coronavirus as coronavirus was first detected in China. This kind of assumption can disrupt social harmony. As SBS can identify such incidences in space and time, it can be used for positive intervention such as providing a right information to communities and providing necessary security to the vulnerable community.

## Discussion

This research shows that social media data analysis is a powerful method for observing social phenomena relevant to an outbreak of an infectious disease such as COVID19. Collecting data through traditional surveys and clinical reports are time-consuming and costly. The process can have a time lag of few weeks between the time of medical diagnosis and the time when the data become available. Unlike traditional methods, social media data analysis is time and cost-effective that can uncover momentum and spontaneity in conversations. This paper showed that social media data analysis can be done systematically to find insights into the underlying problem and can be generalised for a wide range of objectives. For example, this study analysed the discussion dynamics of COVID19 on Twitter from geospatial and temporal context using various methods of volume, sentiment, topic, and impact analysis. These analysis methods were found effective in capturing interesting insights and directly correlated with real-world events.

The overall COVID19 related discussion on Twitter represent more on negative aspects. People were concerned about jobs, economy and isolation in addition to health and safety. For example, there were changes in tweet topics and negative sentiments when the new COVID19 cases were found or death occurs. However, initiatives such as government subsidies made a positive influence. For example, the peaks in positive sentiment occurred during positive initiatives taken by leaders or any positive development in the health care sector. When the spread of new cases started to decline, the number of COVID19 related posts declined and the positive sentiment increased. This analyses show how social media platforms can influence the public’s risk perception, their hope and reliance on different organisational initiatives. It can play an active role in changing the real-world behaviour with an impact on control measures enacted to mitigate an outbreak.

Topic modelling discovered a wide variety of topics in discussion that cover consequences, initiatives, impacts and peoples’ behaviour during this period. Topic analysis provided an understanding of community’s discussion of COVID19 with a reasonable objectivity, precision and generality. With spatio-temporal modelling, we showed how these topics evolved and their significance changed over time. We found that the majority of the COVID19 related discussions have a high concentration around a relatively small number of influential topics. For example, at the beginning, the discussions cenetered on COVID19 outbreak in China, then they progress to COVID19 cases in Australia and health care, to stay home and job loss. SBS further extended the usefulness of topics discovered by understanding the impact of various key concepts in the tweets. For example, topic modelling uncovered racism instances and SBS identified their impact. Our analysis could reveal that COVID19 pandemic created fear and the fear led to racism to thrive that can disproportionately affect marginalised groups.

The findings can help government, emergency agencies, clinicians, health practitioners and caregivers to better utilise social media to understand the public opinion, sentiments, social and mental health issues related to COVID19. Such an understanding will enable proactive decision making for prioritising supports in geo-spatial locations. For example, timely disseminating and updating information related to social issues by the government can contribute to stabilising social harmony.

## Conclusion

We proposed an SNTM model for topic analysis and an INN model for Sentiment Analysis. We rigorously evaluated these two models using several datasets. Then, we applied these two models for analysing a large Spatio-temporal tweet dataset of the Australian Twitter sphere. Additionally, we applied volume analysis, Dynamic Topic Modelling and SBS to obtain insight into COVID19 outbreak in different states and cities of Australia over time. Advanced analysis of social media data related to an ongoing pandemic such as COVID19 is critical to protect public health, maintain social harmony and save lives. By leveraging anonymised and aggregated geo-spatial and temporal data from social media, institutions and organizations can get insights into community discussion to understand and act based on how COVID19 spread is affecting people’s lives and behaviour. Specifically, the government and emergency agencies can use the insights to better understand the public opinion and sentiments to accelerate emergency responses and support post-pandemic management.

Even though we observed that social media data analysis can give very useful insights about ongoing society, there are several limitations of this study. Social media is presenting the opinion of users who use social media so it may not be representing non-social media users. Also, it is an Australia data study, not a world-wide users, and findings only relate to the period chosen.

## Footnotes

1 Location of author or tweet or a location mentioned in the tweet is Australia or any of its cities

2 https://bit.ly/2Z6RUvU.

3 https://spacy.io/usage/linguistic-features.
